# Relationship between Bone Mineral Density and Balance Disorders in Osteoporotic Patients

**DOI:** 10.3389/fbioe.2013.00005

**Published:** 2013-09-19

**Authors:** Fatemeh Radaei, Shahriar Gharibzadeh

**Affiliations:** ^1^Neural and Cognitive Sciences Laboratory, Biomedical Engineering Faculty, Amirkabir University of Technology, Tehran, Iran

**Keywords:** bone mineral density, the center of the mass, bone loss, instability, osteoporotic patients

It is known that due to decreasing bone formation, Bone Mineral Density (BMD) decreases in osteoporotic patients (Bone et al., 2000). In addition, some researchers have shown that osteoporotic patients suffer from balance disorders and increasing risk of falls (Sinaki et al., 2005).

Basically, five factors determine one’s level of stability and mobility: (1) size of the base of support, (2) height of the center of mass, (3) location of the center of mass projection within the base of support, (4) body mass, and (5) friction (Whiting and Rugg, 2005). In some researches, it has been mentioned that height of the center of the mass decreases in osteoporotic patients, but its effect on instability of the patients is not significant, and in osteoporotic cases, there will be a great reduction in body mass, which has a negative effect on their stability (Ludlow, 2006). Researches have not mentioned the change of size of the base of support and friction (first and five factors).

Based on the above-mentioned points, we think that factors 1 and 5 will not change. In addition, we hypothesize that in osteoporotic patients, who have less BMD in comparison to age matched healthy individuals, center of mass of their bones and as the result, center of mass of their body have changed, so the location of their center of mass projection within the base of support changes. Hence, osteoporotic patients have balance disorders while standing or walking and risk of falls is high among them. It is proposed that they wear special shoes in order to compensate the change of center of mass projection to reduce the risk of falls, and their shoes should be provided with more frictional resistance at the interface between the ground and any contact point to increase their stability. Surely experimental researches and clinical trials are needed to validate our hypothesis.
